# The Antioxidant Transcription Factor Nrf2 in Cardiac Ischemia–Reperfusion Injury

**DOI:** 10.3390/ijms222111939

**Published:** 2021-11-03

**Authors:** Ana Mata, Susana Cadenas

**Affiliations:** 1 Centro de Biología Molecular “Severo Ochoa” (CSIC/UAM), 28049 Madrid, Spain; amata@cbm.csic.es; 2 Instituto de Investigación Sanitaria Princesa (IIS-IP), 28006 Madrid, Spain

**Keywords:** cardioprotection, ischemia–reperfusion injury, ischemic conditioning, Nrf2 activators, oxidative stress, reactive oxygen species, redox homeostasis

## Abstract

Nuclear factor erythroid-2 related factor 2 (Nrf2) is a transcription factor that controls cellular defense responses against toxic and oxidative stress by modulating the expression of genes involved in antioxidant response and drug detoxification. In addition to maintaining redox homeostasis, Nrf2 is also involved in various cellular processes including metabolism and inflammation. Nrf2 activity is tightly regulated at the transcriptional, post-transcriptional and post-translational levels, which allows cells to quickly respond to pathological stress. In the present review, we describe the molecular mechanisms underlying the transcriptional regulation of Nrf2. We also focus on the impact of Nrf2 in cardiac ischemia–reperfusion injury, a condition that stimulates the overproduction of reactive oxygen species. Finally, we analyze the protective effect of several natural and synthetic compounds that induce Nrf2 activation and protect against ischemia–reperfusion injury in the heart and other organs, and their potential clinical application.

## 1. Introduction

In addition to producing cellular energy in the form of ATP from fuel oxidation, mitochondria are also an important source of reactive oxygen species (ROS), which play a central role in redox signaling and contribute to oxidative damage across a range of pathologies [1,2,3]. The transcription factor nuclear factor erythroid 2-related factor 2 (Nrf2) is a critical regulator of the cellular stress response. Nrf2 belongs to the cap’n’collar (CNC) family of basic leucine zipper (bZIP) transcription factors [4,5] together with Nrf1 [6], Nrf3 [7], NF-E2 p45 subunit [8], and the more distant related factors BTB domain and CNC homolog 1 and 2 (Bach1 and Bach2) [9]. Nrf2 is widely expressed in mammalian tissues including kidney, liver, heart, lung, brain and skeletal muscle [8,10]. In response to different activation stimuli, Nrf2 translocates from the cytoplasm to the nucleus, where it activates the transcription of its downstream targets by binding to a *cis*-acting enhancer with a core nucleotide sequence of 5′-TGACNNNGC-3′ where N is any nucleotide, termed the antioxidant response element (ARE) or the electrophile response element (EpRE) [11,12]. Nrf2 is believed to control the basal and inducible expression of over 1000 genes involved in antioxidant defense, detoxification, inflammatory response, proteasomal and autophagic degradation, and metabolism [13,14,15], reflecting its multiple cellular functions––from antioxidant defense to protein quality control and metabolism regulation. In line with the focus of the present review, there is substantial evidence for a protective role of Nrf2 in cardiovascular diseases, including atherosclerosis, ischemia–reperfusion (IR) injury, cardiac hypertrophy, heart failure and diabetes (reviewed in [16]), although some harmful effects have also been reported [17,18,19]. For example, Nrf2 activation protects the heart along with other organs from the damage that ensues upon restoration of blood flow to an ischemic tissue, which is known as IR injury. In addition, a number of small molecules, mostly derived from natural products such as sulforaphane, carnosic acid and curcumin, have been identified as Nrf2 activators with demonstrated protection against IR injury in several preclinical models. Herein, we examine the role of Nrf2 in cellular redox homeostasis and the regulation of its transcriptional activity. We also review studies on the protection offered by the aforementioned compounds against cardiac IR injury.

## 2. Nrf2 Signaling and Cellular Redox Homeostasis

Nrf2 activates the expression of genes containing ARE sequences in their promoters, including those coding for glucose 6-phosphate dehydrogenase, 6-phosphogluconate dehydrogenase, malic enzyme 1 and isocitrate dehydrogenase 1, which are all involved in the generation of NADPH, an essential cofactor for antioxidant reactions [20,21,22]. NADH is further used by a myriad of redox reactions, many of which are also regulated by Nrf2. For instance, Nrf2 modulates the expression of critical enzymes involved in the production and use of glutathione (GSH), such as glutamate–cysteine ligase catalytic and modulatory subunits (Gclc, Gclm), glutathione reductase, glutathione peroxidase and several glutathione-S-transferases (GST) [23]. Moreover, proteins of the redoxin family (thioredoxin, thioredoxin reductase, peroxiredoxin and sulfiredoxin), which catalyze redox reactions in different compartments, are controlled by Nrf2 [24,25,26]. It also regulates the expression of genes encoding NAD(P)H:quinone oxidoreductase 1 (NQO1), a flavoprotein that inhibits redox cycling of xenobiotics and maintains the endogenous antioxidants α-tocopherol-hydroquinone and coenzyme Q in their reduced (active) forms, along with heme oxygenase-1 (HO-1), which is involved in the production of the potent physiological antioxidant bilirubin [27]. Additionally, Nrf2 influences cellular elimination of xenobiotics mediated by many phase I/II drug-metabolizing enzymes [13], as well as the multi-drug-resistance-associated transporters (Mrps) [28]. Nrf2 knockout mice provided in vivo evidence that Nrf2 drives the expression of these antioxidant/cytoprotective genes [20,29]. Finally, Nrf2 affects intermediary metabolism, through crosstalk with the pentose phosphate pathway and glycolysis, in addition to increasing the availability of substrates and reducing equivalents for the mitochondrial respiratory chain [30] as well as for mitochondrial DNA (mtDNA) integrity [31]. The finding of an ARE-like element in the proximal promoter of Nrf2 gene (*Nfe2l2*) indicates that it can autoregulate its own transcription [32], leading to positive feedback and amplification of the Nrf2 transcriptional network once it is activated. In summary, Nrf2 increases the cellular defense mechanisms against xenobiotic and oxidative stress through the coordinated expression of numerous antioxidant and detoxification genes. 

## 3. Regulation of Nrf2 Transcriptional Activity

**Figure 1:**
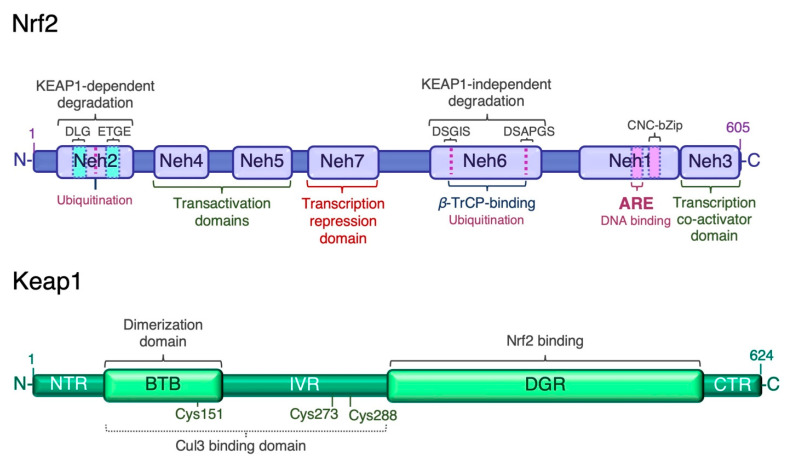
Structure of human Nrf2 and Keap1. The high-affinity ETGE and low-affinity DLG motifs in the Neh2 domain of Nrf2 are bound by the Kelch domain of Keap1 for Nrf2 ubiquitination and degradation. ARE, antioxidant response element; BTB, broad-complex, tramtrack and bric-à-brac domain; CTR, C-terminal region; DGR or Kelch, double glycine repeat domain; IVR, intervening region; Neh, Nrf2-erythroid-derived CNC homology (ECH) domain; NTR, N-terminal region.

Given the wide range of cellular processes controlled by Nrf2, its activity is tightly regulated at multiple levels. Nrf2 has seven Nrf2-ECH homology domains (Neh1–7), which are critical for its activity and repression [13] (Figure 1). The amino-terminal Neh2 domain contains two binding motifs, DLG and ETGE, which mediate binding to the double glycine repeat (DGR) domain of Kelch-like ECH associated protein 1 (Keap1), a negative regulator of Nrf2. A nuclear localization signal (NLS) sequence is localized in this domain. The Neh6 domain is a serine-rich region involved in the negative regulation of Nrf2 stability independently of Keap1. It contains two conserved peptide motifs, DSGIS and DSAPGS, which are recognized by *β*-transducing repeat-containing protein (*β*-TrCP) [33]. *β*-TrCP binds more efficiently to the Neh6 domain after glycogen synthase kinase-3*β* (Gsk-3*β*)-mediated phosphorylation of the DSGIS motif, and promotes the recruitment of the S-phase kinase 1 (Skp1)-Cullin 1 (Cul1)-F-box protein 1 (SCF) ubiquitin ligase complex, leading to proteasomal degradation of Nrf2 [34,35,36,37]. The carboxyl-terminal Neh3 is necessary for transcriptional activation of Nrf2 by recruiting the coactivator chromo-ATPase/helicase DNA-binding protein (CDH) 6. The Neh3 domain contains a second NLS sequence. The Neh1 domain adjacent to Neh3 contains the basic CNC-bZIP region, which is necessary for DNA binding and association with Nrf2 dimerization partners, the small musculoaponeurotic fibrosarcoma (sMaf) proteins [38]. The Neh1 domain contains a nuclear export signal (NES) sequence, and the amino acid sequence of the basic region is highly conserved across a wide range of species [39]. The Neh4 and Neh5 domains are two independent transactivation domains that interact with cAMP response element-binding protein (CREB)-binding protein (CBP) and/or receptor-associated coactivator 3 (RAC3) [40]. The Neh7 domain mediates the repression of Nrf2 transcriptional activity by physical interaction with retinoid X receptor α (RXRα) [41]. The multiple levels of Nrf2 regulation have been recently reviewed [15,42,43]. Transcription mediated by Nrf2 is activated by several transcription factors, which expands the types of stressors that induce Nrf2-target genes. These factors include the aryl hydrocarbon receptor (AhR) [44,45], peroxisome proliferator-activated receptor (PPAR)γ [46,47], nuclear factor-κB (NF-κB) [48,49], specificity protein 1 (Sp-1) [50], p53 [51], c-Jun, c-Myc [52,53], and breast cancer 1 (BRCA1) [54]. As mentioned earlier, Nrf2 also enhances its own transcription [32].

### 3.1. Keap1-Dependent Proteasomal Degradation of Nrf2

**Figure 2:**
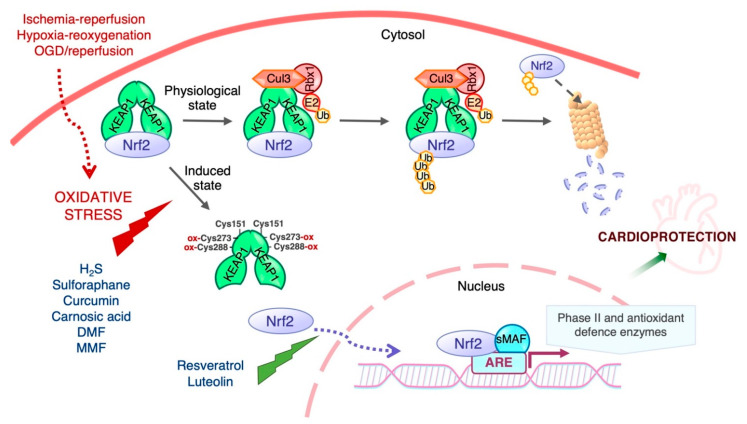
Regulation of Nrf2 transcriptional activity by Keap1. Nrf2 activity and hence the expression of its target genes is maintained at low levels by Keap1 under normal homeostatic conditions, but increases rapidly in response to redox and electrophilic stressors as well as by stimulation by growth factors. Cys151 is required for triggering Nrf2 signaling by activating agents, and Cys273 and Cys288 are functionally important for the sensing of inducers [56]. The biological effects of Nrf2 are exerted through its ability to mediate the induction of genes containing an antioxidant response element (ARE) in their promoter region upon exposure to a broad spectrum of oxidants and eletrophiles.

The transcriptional activity and protein stability of Nrf2 is mainly regulated by its cytosolic inhibitor Keap1, a cysteine-rich protein that contains two major domains, BTB and DGR or Kelch, and three additional domains: the N-terminal region (NTR), the intervening region (IVR), and the C-terminal region (CTR). The BTB domain is critical for Keap1 homodimerization and interaction with the Cul3-Rbx1-E3 ligase complex, while the Kelch domain binds to the DLG and ETGE motifs in the Neh2 domain of Nrf2 (Figure 1). Keap1 contains many cysteine residues (27 cysteines of 624 amino acids in the human homologue) in both the BTB and IVR domains that sense oxidative and/or electrophilic molecules [55,56,57]. Under normal conditions, Keap1 targets Nrf2 for degradation in the cytosol. With a half-life of ~20 min [58], the Keap1-mediated high turnover of Nrf2 keeps Nrf2 levels extremely low to avoid unnecessary gene transcription [59,60]. The prevailing mechanistic model for Nrf2 regulation by Keap1 is the “hinge and latch” model [61]: the ETGE motif of Nrf2 acts as the “hinge” while the DIG motif functions as the “latch”. Keap1 binding to Nrf2 leads to its ubiquitination by the Cul3-Rbx1-E3 ligase, thereby targeting it for proteasomal degradation. Under oxidative stress conditions, oxidative or electrophilic molecules (inducers) react with critical cysteine residues of Keap1, triggering conformational changes that release Nrf2, allowing it to evade Keap1-mediated ubiquitination and degradation. Nrf2 translocates to the nucleus, where it enhances the transcription of Nrf2-driven genes (Figure 2). In addition to oxidative and electrophilic reactions, other types of post-translational modifications of Keap1 have been shown to regulate Nrf2 activity, including ubiquitination and phosphorylation [15]. Likewise, protein kinase C (PKC) phosphorylation of Ser40 in the Neh2 domain of Nrf2 disrupts Keap1-Nrf2 association and promotes Nrf2 activation [62].

### 3.2. Keap1-Independent Regulation of Nrf2

In addition to Keap1-dependent proteosomal degradation of Nrf2, Keap1-independent mechanisms have also been reported. As described above, the Neh6 domain of Nrf2 contains two highly conserved regions that include the DSGIS and DSAPGS motifs, which are recognized by *β*-TrCP. Binding of *β*-TrCP to Nrf2 follows Gsk-3*β*-mediated phosphorylation of the Neh6 domain of Nrf2. *β*-TrCP functions as an adaptor for the SCF E3 ubiquitin ligase complex to regulate proteasomal degradation of Nrf2 [34,35]. Gsk-3*β* action is antagonized by PI3K-PKB/Akt signaling [13,33]. Additionally, Nrf2 activity could potentially be enhanced by some kinases, such as extracellular signal-regulated kinase (ERK), p38 MAP kinase (MAPK), PI3K and PKC, through the inhibition of Gsk-3*β* [63]. Another ubiquitin-dependent system responsible for Nrf2 degradation involves the E3 ubiquitin ligase synoviolin (Hrd1) during liver cirrhosis [64].

Ubiquitylation of Nrf2 at the N-terminal Neh2 and C-terminal Neh3 domains by CR6-interacting factor (CRIF)1 is yet another mechanism by which Nrf2 activity is suppressed [65], whereas Nrf2 acetylation at multiple Lys residues in the Neh1 domain by p300/CBP in response to arsenite-induced stress is activating [66]. Interestingly, Nrf2 can be polysumoylated by small ubiquitin-like modifier (SUMO)-1 and SUMO-2, which target the protein to promyelocytic leukemia nuclear bodies [67]. Within the nucleus, the polysumoylated Nrf2 can be ubiquitylated by the SUMO-specific RING finger protein 4 (RNF4), thereby providing a means of degrading the transcription factor within the nucleus, thus preventing the induction of Nrf2-target genes.

### 3.3. Post-Transcriptional Regulation of Nrf2

Recent studies have revealed important roles of microRNAs (miRNAs, miRs) in the regulation of Nrf2 activity through direct targeting of the Nrf2 mRNA and of mRNAs encoding proteins that control the levels and activity of Nrf2 [68,69,70]. miRNAs are short (approximately 23 nucleotides), single-stranded, non-coding RNAs that regulate gene expression by sequence-specific binding with mRNA molecules and consequent inhibition of translation or degradation [71]. Following transcription, the primary miRNA (pri-miRNA) undergoes several steps of maturation to form precursor miRNA (pre-miRNA), which, upon export to the cytoplasm, is processed to form mature miRNA [72]. Complimentary binding of miRNAs to mRNA targets (most commonly within the 3′-UTR) leads to the recruitment of the RNA-induced silencing complex (RISC) to repress the translation of targeted mRNAs or to induce their degradation [73]. Initial in silico analysis predicted 85 Nrf2-miRNA interactions, with 63 miRNAs that can directly or indirectly regulate Nrf2 [74]. Subsequently, using a more stringent criteria to identify miRNAs that can directly target Nrf2, 16 miRNAs were predicted to target Nrf2: miR-374a, miR-28, miR-708, miR-27b, miR-23a, miR-340, miR-153, miR-507, miR-129, miR-93, miR-144, miR-410, miR-140, miR-129, miR-132, miR-212 [68]. Six of these miRNA species have been confirmed to bind to the 3′-UTR of Nrf2 and to regulate translation of the mRNA of a reporter gene: miR-28 [75], miR-153 [76], miR-507 [77], miR-129 [77], miR-93 [78] and miR-144 [79]. In addition, Nrf2 can be targeted by miR-153, miR-27a, miR-142-5p and miR-144 in neuronal SH-SY5Y cells [80]. Expression of miR-144 is upregulated in aged cerebromicrovascular endothelial cells, reducing Nrf2 expression [81]. Overexpression of miR-144 promotes cell death in retinal pigment epithelium (RPE) while its inhibition potentiates Nrf2-dependent antioxidant signaling and protects against oxidative stress-induced outer retinal degeneration [82]. miR-93 also targets Nrf2 inhibiting cell proliferation and promoting apoptosis in RPE cells [83]. Conversely, Nrf2 regulates miRNAs either increasing or decreasing their expression through direct binding of Nrf2 to promoters of genes coding miRNAs and transcriptional activation [84,85,86,87]. Targeting these miRNAs could be a useful strategy to activate specific Nrf2-mediated signaling avoiding the negative consequences of excessive Nrf2 activation.

## 4. Cardiac Ischemia–Reperfusion Injury

IR injury occurs in many pathological situations including myocardial infarction and stroke, and develops when blood supply is restored to an organ (reperfusion) following a period of poor perfusion (ischemia). Prolonged ischemia triggers a myriad of biochemical and metabolic changes [88]. For instance, the absence of oxygen inhibits oxidative phosphorylation, which causes mitochondrial membrane depolarization, ATP depletion, and inhibition of contractile activity in the myocardium. In this context, cellular metabolism switches to anaerobic glycolysis, resulting in the accumulation of lactate, which leads, in turn, to intracellular calcium overload. While restoration of blood flow to an ischemic organ is essential to prevent irreversible cellular injury, reperfusion per se may augment tissue injury relative to that produced by ischemia alone. During reperfusion, the electron transport chain is reactivated, generating excessive ROS. Other sources of ROS that contribute to reperfusion-induced oxidative stress and represent targets for therapeutic intervention include xanthine oxidase and NADPH oxidase [3,89,90,91,92,93,94,95]. The combined effects of ROS and elevated calcium are believed to lead to the opening of the mitochondrial permeability transition pore (mPTP) in the inner mitochondrial membrane, which plays a critical role in IR injury [96,97,98,99,100]. Regarding the mechanism of mitochondrial ROS production in IR, early reports revealed an increase in succinate in the heart during ischemia and its rapid decrease following reperfusion [101,102]. These observations pointed to mitochondrial superoxide production from complex I driven by reverse electron transfer (RET) from succinate as a key mediator of IR injury. This was confirmed by Chouchani and colleagues using a metabolomics approach and a mitochondria-targeted mass spectrometric hydrogen peroxide probe, MitoB. They showed that succinate-driven RET favors mitochondrial matrix superoxide production from complex I early in reperfusion [103,104,105]. Supporting this analysis, the mitochondria-targeted ubiquinone derivative MitoQ, an established superoxide scavenger [106], has been shown to protect the heart from IR injury [107]. Furthermore, succinate dehydrogenase inhibition by malonate lessens ROS production in both isolated cardiomyocytes and in hearts subjected to IR [103]. However, by monitoring the rapid changes in both NADH/NAD^+^ ratio and flavoprotein redox state during reperfusion of the heart using real-time surface fluorescence, Andrienko et al. found that these conditions were unfavorable for RET [108,109]. Using a Langendorff perfused heart model, the authors failed to demonstrate that MitoQ was cardioprotectiove, even though it delayed mPTP opening, inhibiting the generation of mitochondrial superoxide during reoxygenation and improving cell survival in isolated cardiomyocytes subjected to hypoxia followed by reoxygenation [110]. The authors also proposed that the rapid loss of succinate upon reperfusion could be due to its efflux rather than to its oxidation by succinate dehydrogenase [109]. Nevertheless, inhibition of succinate dehydrogenase during reperfusion slows the decline in succinate and limits infarct size, supporting the notion that succinate oxidation may contribute to cardiomyocyte death when oxygen becomes available [111].

## 5. Role of Nrf2 in Ischemia–Reperfusion Injury

Given that oxidative stress-related insults activate Nrf2, which in turn induces the expression of an array of cytoprotective genes, the protective role of Nrf2 against IR injury has been extensively explored in the heart and other organs. Myocardial reperfusion after ischemia induces de novo synthesis of Nrf2, which protects against IR injury [112]. Likewise, reperfusion-specific activation of Nrf2 has been reported in renal epithelial cells exposed to hypoxia/reoxygenation [113]. In this latter study, attenuating ROS overproduction in reoxygenation with the antioxidant N-acetyl cysteine (NAC) suppressed Nrf2 activation, pointing to ROS as signaling mediators of Nrf2. Along the same line, activation of the Nrf2 pathway by miRNAs protects rat and mouse cardiomyocytes against in vitro IR or oxygen and glucose deprivation (OGD) followed by reperfusion [114,115,116]. We have shown that Nrf2 expression is elevated during reperfusion in isolated perfused mouse hearts (Langendorff system) [117]. Data from Xu et al. using a model of left anterior descending (LAD) coronary artery occlusion [112], and our own data in isolated perfused mouse hearts (unpublished), revealed that Nrf2-knockout mice present with increased infarct area as determined by 2,3,5-triphenyltetrazolium chloride (TTC) staining, and enhanced cardiac troponin I [112] or creatine kinase (our data) activity relative to wild-type mice, supporting a protective role for Nrf2 against cardiac IR injury. In addition, administration of sub-lethal concentrations of the lipid peroxidation product 4-hydroxy-2-nonenal (4-HNE) induced Nrf2 activation in mouse cardiomyocytes [118,119], which protected them against glucose-free anoxia followed by reoxygenation [118]. Likewise, intravenous administration of 4-HNE activated Nrf2 in the heart, increasing glutathione content, and improved the functional recovery of the left ventricle following IR in Langendorff-perfused hearts from wild-type but not from Nrf2-knockout mice [118]. Nrf2 activation has also been reported to protect the murine heart against pathological cardiac hypertrophy and heart failure by suppressing oxidative stress [120]. In murine heterotopic heart transplant models, Nrf2 was shown to inhibit NF-κB activation and protect against IR injury, which was mediated by its antioxidant activity, and suppressed the subsequent development of cardiac allograft vasculopathy [121]. Of note, contrary to the great majority of the studies showing beneficial effects of Nrf2 activation against IR injury, Erkens et al. reported that global Nrf2 gene knockout attenuates myocardial IR injury and dysfunction in mice, which the authors suggested was most likely due to cardioprotection by endothelial nitric oxide synthase (eNOS)-derived nitric oxide [17].

Several miRNAs modulate IR injury by regulating the Nrf2 pathway (reviewed in [122,123,124]). Thus, upregulation of miRNA-153 inhibits Nrf2/heme oxygenase-1 and induces ROS production and apoptosis in cardiomyocytes after OGD followed by reoxygenation [115] and in a rat model of IR [116]. Overexpression of miR-210 in cardiomyocytes reduces ROS production and cell death, while downregulation of miR-210 increases ROS levels after hypoxia/reoxygenation [125]. Although not investigated in this study, it seems likely that the effects of miR-210 are mediated by Nrf2. The dual blockade of miR-24-3p and miR-145-5p leads to synergistic upregulation of shared target protective antioxidant genes and inhibition of ROS production in human umbilical vein endothelial cells (HUVECs) after hypoxia/reoxygenation [126]. Interestingly, miR-24-3p [114] and miR-200a [127] target Keap-1, consequently activating Nrf2, thus protecting cardiomyocytes from IR injury [114] or hypoxia-induced apoptosis [127].

In addition to heart protection, Nrf2 also protects other organs against IR injury. For instance, in a murine model of middle cerebral artery occlusion (MCAO) and reperfusion, Nrf2-knockout mice showed a larger hemisphere infarct volume than wild-type mice along with a greater neurological deficit [128]. Consistent with a protective role of Nrf2, IR-mediated cortical damage and sensorimotor deficit in rats was ameliorated by treatment with *tert*-butylhydroquinone, an Nrf2 activator [129]. Nrf2-knockout mice have also been reported to show more severe retinal damage after IR (high intraocular pressure) than wild-type mice, with Nrf2 believed to mediate the neuroprotective effects of histone deacetylase inhibitors in retinal IR injury [130]. Likewise, a higher number of apoptotic cells was reported in the skeletal muscle of Nrf2-knockout mice when compared with wild-type counterparts after hind-limb IR injury [131]. In the context of liver IR injury, Nrf2 deficiency in mice enhanced tissue damage, impaired ARE-regulated gene signaling, disturbed the redox state and aggravated tumor necrosis factor α mRNA expression [132]. Similarly, in a murine model of warm hepatic IR injury, Nrf2 deficiency exacerbated hepatic injury, whereas hepatocyte-specific Nrf2 overactivation provided protection [133]. Nrf2 activation has been shown to limit macrophage and neutrophil trafficking, proinflammatory cytokine programs and hepatocellular necrosis or apoptosis in IR-stressed mouse liver while increasing antiapoptotic functions, whereas ablation of Nrf2 signaling exacerbated IR-induced liver inflammation and damage [134]. At the molecular level, Nrf2 activation augmented HO-1 expression and promoted PI3K-Akt signaling while suppressing forkhead box O (FoxO)1 signaling, leading to diminished expression of TLR4 proinflammatory mediators [134]. Nrf2 also prevented oxidative injury in a murine model of IR-stressed orthotopic liver transplantation, limiting hepatic inflammatory responses and hepatocellular apoptosis in a PI3K-dependent manner [135]. Nrf2 deficiency is known to enhance susceptibility to both ischemic and nephrotoxic acute kidney injury, as reflected in worsened renal function, histology, vascular permeability and survival in Nrf2-knockout mice compared with wild-type mice after IR, in addition to increasing proinflammatory cytokine and chemokine expression [136]. The knockout mice were also more susceptible to cisplatin-induced nephrotoxicity, which was blunted by NAC pre-treatment, partly mitigating Nrf2 deficiency. These results agree with the finding that global and tubular-specific Nrf2 activation enhances gene expression of antioxidant and NADPH synthesis enzymes, including glucose-6-phosphate dehydrogenase, and ameliorates both the initiation of injury in the outer medulla and the progression of tubular damage in the cortex [137]. Taken together, the data obtained in different organs identify Nrf2 as a potential therapeutic target in IR injury.

## 6. Involvement of Nrf2 in Protective Ischemic Conditioning

Ischemic conditioning is a phenomenon where brief episodes of ischemia confer protection against injury from subsequent prolonged ischemia. In patients, this cardioprotective strategy can be applied to the heart directly before surgical intervention (ischemic preconditioning, IPC) or after the injury (ischemic postconditioning); it can also be applied to other parts of the body, such as a limb (remote pre- or postconditioning) [138]. Although the phenomenon was first described 35 years ago [139], the mechanism of protection is still not fully understood. Recent studies have investigated the role of Nrf2 in IPC, as the activation of the Nrf2/ARE pathway could be responsible for the induction of antioxidant enzymes caused by IPC, contributing to the late cardioprotection. For example, in H9c2 cells, a cell line derived from embryonic rat heart, IPC induced the expression of Nrf2-dependent antioxidant enzymes, which conferred late protection against the resultant oxidative stress from hypoxia/reoxygenation [140]. Similarly, in an isolated rabbit heart model, IPC significantly induced activation of Nrf2 and downstream antioxidant genes HO-1 and manganese superoxide dismutase (MnSOD), and led to a better recovery of the isolated rabbit heart after IR [141]. These latter effects were mediated by the activation of protein kinase C (PKC), which induces nuclear translocation of Nrf2 and enhances endogenous antioxidant defenses. PKC was also reported to mediate Nrf2 phosphorylation and activation in an in vivo rat model of IR during post-conditioning, which resulted in cardioprotection [142]. As might be expected, Nrf2-knockout mice lose IPC-mediated cardioprotection and exhibit increased infarct size in response to IR [112]. Indeed, several studies have shown that Nrf2 is activated in IPC and induces antioxidant enzymes and antiapoptotic proteins to protect against subsequent IR [112,143,144]. In addition, Nrf2 was found to mediate hepatoprotection afforded by remote ischemic conditioning in a murine model of hemorrhagic shock/resuscitation [145]. Similarly, in a rat model of renal ischemia, IPC and the phytochemical sulforaphane (see below), or a combination of both, activated Nrf2 and increased the expression of its target genes, improving renal function and reducing the expression of inflammatory cytokines [146]. Hepatic IR in rats and mice induces the kidney expression of HO-1, an enzyme with antioxidant and anti-inflammatory functions, via Nrf2, which protects the kidney from remote organ damage after liver IR [147].

In a rat brain model of ischemic stroke, sulforaphane preconditioning prevented neurological dysfunction by activating the Nrf2/ARE pathway [148]. In addition, Nrf2 mediated the IPC-induced protection in astrocytes in an OGD model of cerebral ischemia and in cultured astrocytes derived from wild-type and Nrf2 knockout mice, which can impact the ischemic tolerance of neurons [149]. Similarly, mild oxidative stress insults, including subtoxic H_2_O_2_, strongly activated Nrf2/ARE-dependent gene expression in murine astrocytes, contributing to neuroprotective IPC [150]. IPC was shown to protect the murine blood–brain barrier against ischemic injury by generation of endogenous electrophiles and activation of the Nrf2 pathway through inhibition of Keap1 and Gsk-3*β*-dependent Nrf2 degradation [151]. Finally, IPC provided long-lasting neuroprotection against ischemic brain injury and post-stroke cognitive dysfunction both in primary rodent cortical neurons subjected to OGD preconditioning and in a model of transient MCAO in wild-type but not in Nrf2-knockout mice [152].

## 7. Activators of Nrf2 and Their Protective Role against Ischemia–Reperfusion Injury

Many natural and synthetic compounds have been identified as Nrf2 activators and have been tested for pharmacological potential, with several showing protective effects against IR injury mainly in the heart, brain and kidney (Table 1). Pharmacological activation of Nrf2 is a promising therapeutic approach for several chronic diseases including neurodegenerative, cardiovascular and metabolic diseases [153,154,155]. Some of these protective compounds induce chemical modification of the sensor cysteines of Keap1, blocking the Keap1-dependent Nrf2 degradation, and allowing de novo synthesized Nrf2 accumulate, translocate to the nucleus and initiate transcription of its downstream target genes [16].

Hydrogen sulfide (H_2_S) is a gaseous signaling molecule produced endogenously in different cells and tissues by the enzymes cystathionine γ-lyase (CGL or CSE) and cystathionine β-synthase (CBS) [157,185,186]. Physiological concentrations are cytoprotective through mechanisms related to ROS scavenging, regulation of cell growth, relaxation of blood vessels and inhibition of leukocyte–endothelial cell interaction [157,186]. Exogenous administration and endogenous overexpression of H_2_S have been shown to protect the heart against IR injury by limiting infarct size and cardiomyocytes apoptosis [187,188,189] through neutralizing ROS overproduction in the peri-infarct zone (the area most affected by reperfusion). The mechanism involved was mediated by the upregulation of protective enzymes by Nrf2. In this context, Calvert and colleagues reported that H_2_S induced the nuclear localization of Nrf2 and increased the protein expression of phase II enzymes, whereas it offered no protection against myocardial IR in Nrf2-deficient mice [156,157]. Similar results have been observed to occur in the brain of mice treated with H_2_S before cerebral IR and in kidney. In this case, sodium hydrosulfide (NaHS), an exogenous H_2_S donor, was found to alleviate tissue injury and inflammation by activating the Nrf2 pathway [158,159]. Using mouse embryonic fibroblasts isolated from CSE-knockout mice, Yang et al. showed that H_2_S inhibits Keap1 by S-sulfhydration of Cys151, resulting in a conformational change in Keap1 that releases Nrf2 [185].

The isothiocyanate sulforaphane is a natural antioxidant compound found in cruciferous vegetables, and is a strong inducer of phase II (biotransforming) enzymes [190,191]. Talalay and Zhang were the first to isolate sulforaphane from broccoli (*Brassica oleracea*) [191] and to demonstrate its cancer protective properties [192]. Sulforaphane has been found to have protective effects against IR injury, degenerative diseases and cancer in preclinical studies [154,193,194]. For instance, sulforaphane has cardioprotective effects in the setting of cardiac injury by preserving cardiac function, decreasing infarct size, oxidative stress and inflammation and activating several kinases and phase II enzymes (glutathione, glutathione reductase, GST, thioredoxin reductase, NQO1) in in vivo and in vitro IR models [160,195,196]. This protective effect has also been investigated in other tissues, particularly in the brain, where sulforaphane pretreatment is neuroprotective [197]. Indeed, sulforaphane has been shown to reduce cerebral infarct volume in a neonatal hypoxia-ischemia brain rat model [161] and in a focal ischemia rat model with common carotid or middle cerebral artery occlusion and reperfusion, and to reduce cytotoxic oxygen radicals in cultured mouse brain microvascular endothelial cells [148,162,163,164]. Of note, the hybrid compound of melatonin and sulforaphane ITH12674 shows improved neuroprotective effects in brain ischemia [198]. Renal damage by IR has also been investigated as a potential target for sulforaphane treatment [197]. Yoon et al. found that sulforaphane protected HK2 cells against hypoxia/reoxygenation damage and improved the renal dysfunction generated by IR in rat kidneys [165]. Importantly, sulforaphane was found to protect both rat kidney [199] and heart [200] after experimental transplantation. These protective properties of sulforaphane are due to nuclear Nrf2 translocation and activation of endogenous oxidative defenses. Mechanistically, it has been proposed that sulforaphane acts similar to H_2_S, oxidizing Cys151 of Keap1 and releasing Nrf2 [16,160,161,165,190,194]. Sulforaphane can also activate the MAPK and PI3K/Akt pathway to phosphorylate Nrf2 and induce its nuclear inclusion [201,202,203]. Clinical trials of sulforaphane (broccoli sprout extract) administration have shown reduced fasting blood glucose and glycated hemoglobin [204], in addition to reduced oxidative stress [205] and favorable effects on lipid profiles and oxidized-LDL/LDL ratio as risk factors for cardiovascular disease [205] in type 2 diabetic patients.

The polyphenolic diterpene carnosic acid, derived from the rosemary plant, has various protective biological properties, including antimicrobial, neuroprotective, anti-inflammatory, anti-cancer and free radical scavenging [167,168]. Carnosic acid was found to be cardioprotective in a H9c2 cardiomyocyte in vitro IR model by diminishing ROS overproduction and improving mitochondrial dysfunction [206]. Likewise, in rodent models of isoproterenol-induced myocardial injury, carnosic acid ameliorated heart dysfunction by activating antioxidant defense system through the Nrf2 pathway [166,167]. According to other studies, carnosic acid has also neuroprotective properties, decreasing infarct size in the brain of mice after MCAO/reperfusion injury and activating Nrf2 in rat PC12h cells [168]. Similar to the aforementioned compounds, carnosic acid stabilizes Nrf2 by S-alkylation of targeted cysteines on Keap1, blocking Nrf2 ubiquitination and degradation, and allowing Nrf2 nuclear accumulation [168,207]. 

Curcumin is a phenolic compound extracted from the rhizome of *Curcuma longa* and is commonly used in Asia as a spice and food additive [208,209]. Curcumin has several antioxidant anti-inflammatory and anti-cancer properties by virtue of its ROS scavenging capacity, and also induces the up-regulation of cytoprotective and antioxidant proteins [169,172,201,208,209]. Curcumin has beneficial effects on infarct size and inflammation in several rat brain global and focal ischemia models, thus demonstrating its neuroprotective role [170,171,172,209,210,211]. Likewise, a curcumin-supplemented diet generated neuroprotection in a gerbil ischemia model [212]. A downside to the use of curcumin as a potential cardioprotectant, however, is that it is poorly absorbed by the digestive system and is quickly metabolized [213]. In primary cultures of rat cortical neurons with OGD/reperfusion and in an MCAO rat model, pre- and post-treatment with curcumin protected against the consequences of IR by increasing the abundance of Nrf2 in the nucleus [169,170,171,172]. Other studies have demonstrated a hepatoprotective role of curcumin against IR damage in rats [173]. There is also evidence of cardioprotection after acute myocardial ischemia in rats [214]. Curcumin has been further studied using analogues (14p) synthesized for greater stability and bioavailability, which act as antioxidants in cardiac H9c2 cells treated with H_2_O_2_ and limit IR-mediated injury in mice with LAD coronary artery occlusion by activating Nrf2. Mechanistically, Keap1 thiol groups are modified by curcumin, which releases Nrf2 [171,201]. A number of clinical trials have shown beneficial effects of dietary supplementation with curcumin in patients with type 2 diabetes [215,216,217,218,219], including positive effects on fasting blood glucose, weight, atherogenic risk and severity of sensorimotor polyneuropathy.

Luteolin is a flavonoid found in many vegetables and fruits commonly used in traditional Chinese medicine because of its anti-oxidative, anti-inflammatory and anti-carcinogenic activities [220,221]. This compound has been tested against myocardial IR injury in diabetic rats. Luteolin was found to act directly as a ROS scavenger and to enhance eNOS-mediated S-nitrosylation of Keap1 [174,175]. Plant extracts with high luteolin content also produce neuroprotection against cerebral IR injury [220]. Luteolin-mediated protection against other tissue damage situations, such as traumatic brain injury or hepatotoxicity, is promoted at least partly by Nrf2 translocation to the nucleus and induction of antioxidant genes [220,221]. Luteolin has also been reported to induce Nrf2-driven HO-1 expression through the activation of the ERK signaling pathway [222,223], and to protect the heart from mercuric chloride (HgCl_2_)-induced cardiac damage via PI3K/Akt activation [221].

Another natural Nrf2 activator is resveratrol, a polyphenolic phytochemical biosynthesized by some plants including grapes, peanut skins and berries. Resveratrol has anti-inflammatory, anti-apoptotic, anti-cancer and oxidative stress-reducing activities. Research on resveratrol began with the “French Paradox”, which describes improved cardiovascular outcomes in French people despite a high dietary intake of fats [177,201,224]. The cardioprotective effect of resveratrol has been studied using in vivo (LAD coronary artery occlusion rat model), ex vivo (isolated perfused rat hearts in Langendorff system) and in vitro (H9c2 cells) IR models, where it has been shown to decrease ROS production and ameliorate infarct size and cardiac dysfunction [176,225]. Cheng and colleagues reported that Nrf2 was key for resveratrol-mediated cardiac protection as rats administered with resveratrol before reperfusion showed increased activity of antioxidant enzymes and enhanced levels of Nrf2 and HO-1 in a model of LAD coronary artery occlusion [176]. In addition, it has been shown that resveratrol protects kidney against IR injury through Nrf2/Keap1 up-regulation, alleviating renal dysfunction [177]. Unlike other Nrf2 activator compounds, resveratrol does not act on Keap1, but instead activates the ERK kinase [226,227] and PI3K/Akt [228,229,230] pathways to phosphorylate Nrf2 and protect against oxidative stress. Although no antioxidant or anti-inflammatory effect of resveratrol was found in non-dialyzed chronic kidney disease patients [231], other clinical trials have demonstrated decreased cardiovascular risk [232] and antidiabetic and antioxidant effects [233] after resveratrol supplementation.

In addition to natural compounds, synthetic compounds have also been studied in IR injury models, including fumaric acid derivatives (FADs), specifically dimethyl fumarate (DMF) and its primary metabolite monomethyl fumarate (MMF). Both are fumaric acid diesters clinically used to treat psoriasis and multiple sclerosis, and both have several biological properties (anti-oxidative stress, anti-apoptotic and immunomodulatory) and are well tolerated with minimal adverse effects [180,234]. DMF has been recognized as a potent agent against oxidative injury in cardiomyocytes cultured in an anaerobic chamber or exposed to OGD followed by reoxygenation through Nrf2 activation [180,234]. Furthermore, Ashrafian et al. showed that both endogenous fumarate accumulation and treatment with exogenous fumarate could protect the heart in a perfused model of myocardial infarction in wild-type mice but not in Nrf2-knockout mice [179]. Both MMF and DMF have also been reported to protect the brain against IR damage by reducing infarct volume, attenuating intracellular oxidative stress and improving the neurological deficits in subacute stages and promoting the Nrf2 expression in MCAO/reperfusion and cerebral HI/reperfusion in mouse wild-type models but not in Nrf2-knockout models [181,182]. The protective role of DMF has also been described in hepatic and intestinal IR lesions [173,183]. Fumaric acids activate Nrf2 by modifying the Cys151 of Keap1, as in the case of H_2_S and sulforaphane, releasing Nrf2 to activate the phase II enzyme transcription in the nucleus [179,181,234]. Although MMF has been used in some studies in brain and retina neuroprotection against IR [181,184] as it is the main bioactive metabolite of DMF, it has been shown that the two compounds have different effects, with DMF being a stronger Nrf2 activator [184].

In summary, many phytochemicals and related compounds activate Nrf2 to increase phase II detoxifying enzymes and other cytoprotective proteins that play significant roles not only in cardioprotection but also in the prevention of IR injury in other organs.

## 8. Concluding Remarks and Future Perspectives

The Nrf2/Keap1 pathway plays a key role in the defense against oxidative stress and the maintenance of redox homeostasis. The damage associated with reperfusion of ischemic tissues (IR injury) is mainly mediated by the excessive production of ROS. The protective capacity of the activation of the Nrf2/Keap1 pathway against IR injury in the heart and other organs has been demonstrated in a number of cell lines and animal models. The most compelling evidence of the protective effect of Nrf2 activation against IR injury is the loss of protection in Nrf2-knockout mice. Many natural and synthetic compounds have demonstrated significant efficacy on activating Nrf2 and, consequently, on inducing genes that collectively regulate much of the endogenous defense system, enhancing cell survival. Some of these compounds are commercially available as dietary supplements. By virtue of their capacity to activate Nrf2, they protect against IR damage. However, the clinical potential of these compounds in protecting of the heart and other organs during reperfusion needs careful evaluation in future clinical trials.

## Figures and Tables

**Table 1:** Natural and synthetic activators of Nrf2 and their effects on IR injury. The Table shows the compound administered and the models used, and describes the outcomes of the study. ARE, antioxidant response element; CCA, common carotid artery; CGL, cystathionine γ-lyase; *Fh1*, fumarate hydratase; H/R, hypoxia/reoxygenation; IR, ischemia–reperfusion; ISO, isoproterenol; LAD, left anterior descending; LCA, left coronary artery; MCAO, middle cerebral artery occlusion; OGD, oxygen and glucose deprivation; ROS, reactive oxygen species.

Compound	Administration and Dose	Model	Outcome	Reference
Hydrogen sulfide	- NaHS intracardiac injection (100 μg/kg) at reperfusion and i.v. for 7 days	- Cardiac-specific transgenic mice overexpressing CGL with LCA occlusion	- Decreased oxidative stress - Attenuation of mitochondrial dysfunction and cardiac injury	[156]
Hydrogen sulfide	- NaHS i.v. injection (100 μg/kg) 24 h before IR	- Cardiac-specific transgenic mice overexpressing CGL and Nrf2-KO mice with LCA occlusion	- Antioxidant, antiapoptotic signaling - Nrf2 nuclear accumulation - Strong cardioprotection	[157]
Hydrogen sulfide	- Inhalation (air mixed with H_2_S at 40 ppm for 8 h) for 7 days before IR	- MCAO in Nrf2-KO mice	- Prevention of abnormal neurological function, inflammation and oxidative injury	[158]
Hydrogen sulfide	- NaHS i.p. injection (50 μmol/kg) before IR	- Renal IR model in Nrf2-KO mice	- Alleviation of inflammatory stress, cell apoptosis and renal injury	[159]
Sulforaphane	- Single injection in the left ventricle cavity (500 μg/kg) before IR	- Proximal LCA occlusion in rats	- Decreased oxidative stress and inflammation - Protective response in hearts	[160]
Sulforaphane	- Single i.p. injection (5 mg/kg) 30 min before hypoxia-ischemia	- Neonatal rat brain hypoxia-ischemia model	- Decreased abundance of apoptotic cells and cytotoxic oxygen radicals - Reduction of infarct volume	[161]
Sulforaphane	- Single i.p. injection (5 mg/kg) 1 h before IR	- CCA and MCAO in rats	- Improves redox-sensitive defenses in the brain - Limits the infarct volume and neurological deficits	[148,162]
Sulforaphane	- Single i.p. injection (5 mg/kg) 1 h before IR - In culture medium (2.5 μM) for 1–4 h	- MCAO in rats - bEnd.3 murine cells	- Increase Nrf2 nuclear accumulation before the infarct - Protects the brain against oxidative damage	[163]
Sulforaphane	- In culture medium (2.5 μM) for 24 h before H/R	- Hyperoxic and normoxic preconditioning and then H/R in bEnd.3 murine cells with Nrf2 silenced by siRNA	- Protection against the generation of ROS	[164]
Sulforaphane	- In culture medium (1-20 μM) for 12 h before H/R - Single i.v. injection (500 μg/kg) 24 h before IR	- H/R in HK2 human kidney cells- Renal IR rat model	- Cytoprotection against H/R toxicity - Reduction of renal dysfunction and injury	[165]
Carnosic acid	- Oral (50 mg/kg) for 5 days	- ISO-induced myocardial injury in rats	- Anti-inflammatory, antioxidant and antiapoptotic effect - Reduction in myocardial injury	[166]
Carnosic acid	- Oral (50 mg/kg) for 12 days	- ISO-induced myocardial stress in mice	- Reduction in oxidative stress, apoptotic status - Abolition of ISO-induced myocardial stress	[167]
Carnosic acid	- Single i.p. injection (1 mg/kg) before IR - In culture medium (10 μM) for 20 h	- MCAO/reperfusion in mice - PC12h cells with Nrf2 dominant-negative constructs	- Induction of phase II enzymes - Neuroprotection	[168]
Curcumin	- In culture medium (10 μM) for 24 h before OGD	- OGD model in rat cortical neurons	- Protection of neurons against cell damage - Activation of Nrf2/ARE	[169]
Curcumin	- Single i.p. injection (300 mg/kg) 30 min after ischemia	- MCAO/reperfusion in rats	- Antioxidation, anti-inflammatory and antiapoptotic - Reduction in brain edema and neurological dysfunction	[170]
Curcumin	- Single i.p. injection (50, 100 mg/kg) 15 min after ischemia	- MCAO in rats (permanent focal ischemia)	- Decreased infarct volume and improved brain edema	[171]
Curcumin	- Single i.p. injection (300 mg/kg) 1 h after IR - In culture medium (2.5–25 μM) for 24 h after OGD	- MCAO/reperfusion in rats - OGD/reoxygenation model in rat cortical neurons	- Reduction in infarct size and oxidative stress levels - Improved cell survival	[172]
Curcumin	- Oral (400 mg/kg) for 14 days before IR	- Hepatic IR in a rat model with vascular clamping	- Attenuation of inflammatory response - Improvement in hepatocyte proliferation and liver protection	[173]
Luteolin	- Intragastrical (100 mg/kg/day) for 2 consecutive weeks	- Isolated perfused rat heart (Langendorff) in a diabetic rat model	- Attenuation of cardiac injury, improved cardiac function and myocardial viability - Activation of Nrf2 antioxidative functions	[174,175]
Resveratrol	- Single i.v. injection (100 μmol/L) 5 min before reperfusion	- LAD coronary artery occlusion in rats	- Antioxidant and anti-inflammatory effects - Reduction in infarct area and improvement in cardiac function	[176]
Resveratrol	- Single intragastrical administration (0.23 μg/kg) 30 min before IR	- Renal IR in a rat model with vascular clamping	- Inhibition of inflammatory response - Reduction in oxidative stress and apoptosis - Renal protection	[177]
Curcumin analogue 14p	- Oral (10, 100 mg/kg) for 7 days before IR	- LAD coronary artery occlusion in mice model	- Reduction in oxidative stress and myocardial apoptosis- Decreased infarct size	[178]
Dimethyl fumarate	- Oral (15 mg/kg) twice daily for 5 days	- IR model in perfused hearts from *Fh1* KO mice - Coronary artery ligation model in Nrf2-KO mice	- Increase of ARE gene expression - Reduction in myocardial infarct size	[179]
Dimethyl fumarate	- In culture medium (5–40 μM) for 24 h before OGD	- H9c2 rat cells cultured in an anaerobic chamber with glucose-free DMEM then in control conditions	- Reduction in ROS production, improvement in cellular viability and antiapoptotic effect	[180]
Dimethyl fumarate	-Oral (30, 45 mg/kg) twice daily for 7 days	- MCAO/reperfusion model in Nrf2-KO mice	- Reduction in neurological deficits - Decreased infarct volume, brain edema and cell death	[181]
Dimethyl fumarate	- Oral (100 mg/kg) for 7 days before hypoxia-ischemia	- Cerebral hypoxia-ischemia mouse model in Nrf2-KO mice	- Reduction in infarct size, brain edema and hippocampal neuronal degeneration - Activation of Nrf2 pathway	[182]
Dimethyl fumarate	- Oral (25 mg/kg) for 14 days before IR	- Hepatic IR in a rat model with vascular clamping	- Induction of antioxidant enzyme expression via Nrf2 - Liver protection	[173]
Dimethyl fumarate	- Oral (15, 25 mg/kg) for 14 days before IR	- Superior mesenteric artery occlusion/reperfusion rat model	- Reduction in oxidative stress and inflammatory response - Intact mucosa	[183]
Monomethyl fumarate	- Single i.p. injection (50 mg/kg) 2 days before IR and daily after IR	- Retinal IR with intraocular pressure increases and restoration model in Nrf2-KO mice	- Increase of Nrf2-regulated antioxidative gene expression - Inhibition of inflammatory gene expression - Decreased neuronal cell loss and improved retinal function	[184]
